# A deep learning approach for predicting severity of COVID-19 patients using a parsimonious set of laboratory markers

**DOI:** 10.1016/j.isci.2021.103523

**Published:** 2021-11-27

**Authors:** Vivek Singh, Rishikesan Kamaleswaran, Donald Chalfin, Antonio Buño-Soto, Janika San Roman, Edith Rojas-Kenney, Ross Molinaro, Sabine von Sengbusch, Parsa Hodjat, Dorin Comaniciu, Ali Kamen

**Affiliations:** 1 Siemens Healthineers, Digital Technology and Innovation, 755 College Road East, Princeton, NJ 08540, USA; 2 Emory University School of Medicine WMB, 1010 Woodruff Circle, Suite 4127, Atlanta, GA 30322, USA; 3 Siemens Healthineers, Laboratory Diagnostics, 511 Benedict Avenue, Tarrytown, NY 10591, USA; 4 Jefferson College of Population Health of Thomas Jefferson University, 901 Walnut Street, Philadelphia, PA 19107, USA; 5 Department of Laboratory Medicine, Hospital Universitario La Paz, Madrid, Spain; 6 Department of Pathology and Genomic Medicine, Houston Methodist Hospital, 6565 Fannin Street, Houston, TX 77030, USA

**Keywords:** Classification Description: Virology, Diagnostics, Artificial intelligence, Machine learning

## Abstract

The SARS-CoV-2 virus has caused tremendous healthcare burden worldwide. Our focus was to develop a practical and easy-to-deploy system to predict the severe manifestation of disease in patients with COVID-19 with an aim to assist clinicians in triage and treatment decisions. Our proposed predictive algorithm is a trained artificial intelligence-based network using 8,427 COVID-19 patient records from four healthcare systems. The model provides a severity risk score along with likelihoods of various clinical outcomes, namely ventilator use and mortality. The trained model using patient age and nine laboratory markers has the prediction accuracy with an area under the curve (AUC) of 0.78, 95% CI: 0.77–0.82, and the negative predictive value NPV of 0.86, 95% CI: 0.84–0.88 for the need to use a ventilator and has an accuracy with AUC of 0.85, 95% CI: 0.84–0.86, and the NPV of 0.94, 95% CI: 0.92–0.96 for predicting in-hospital 30-day mortality.

## Highlights

• Algorithm using 9 laboratory markers & age may predict severity in patients with COVID-19
• Model was trained and tested on a multicenter sample of 10,937 patients
• Algorithm can predict ventilator use (NPV, 0.86) and mortality (NPV, 0.94)
• High NPV suggests utility as an adjunct to aid in triaging of patients with COVID-19

## Graphical abstract

**Figure:**
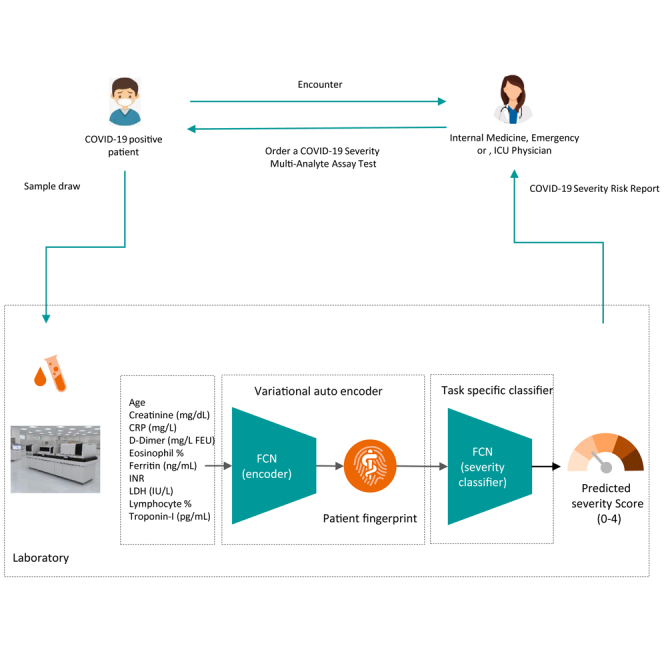

## Introduction

The COVID-19 pandemic has emerged as a worldwide health challenge with an overwhelming burden on healthcare systems, emphasizing the need for effective management of rapidly deteriorating patients constrained by limited clinical resources(Erika et al., 2020; Ranney et al., 2020). Clinical presentation of COVID-19 ranges from mild symptoms to various levels of respiratory distress and/or multi-organ system failure and death(Grant et al., 2020). Effective clinical management of these patients is dependent on early stratification of patients who have a higher likelihood of deteriorating(Marini and Gattinoni, 2020). For example, it has been suggested that a subset of patients with higher likelihood of severity detected during the first encounter and presentation could benefit from immunomodulatory treatments in addition to purely antiviral treatment strategies (Meyerowitz et al., 2020).

Recently, there has been a proliferation of studies proposing prediction models based on various clinical parameters aimed at early stratification of deteriorating patients (Abdulaal et al., 2020; Aktar et al., 2021; Gao et al., 2020; Yan et al., 2020). These approaches are primarily based on traditional machine learning approaches (Aktar et al., 2021) such as support vector machines (SVM) (Gao et al., 2020), random forests (RF) (An et al., 2020), or deep neural networks based (DNN) (Li et al., 2020) methods specifically when it comes to analyzing X-ray or computed tomography (CT) images (Lassau et al., 2021). Despite these advances, we have yet to see a practical system, which could be used universally with an evidence of generalizability to help with early identification of patients, who develop severe clinical trajectory. The underlying reasons could be summarized by two main factors. First, most of proposed models are trained and validated based on small regional cohorts of less than 10,000 patients(An et al., 2020; Assaf et al., 2020; Burdick et al., 2020; Cabitza et al., 2020; Gao et al., 2020; Li et al., 2020). This potentially introduces biases in the results, which in turn hinders generalization and replication of findings to patients from different healthcare systems. Second, there has been a focus to be as comprehensive as possible and to use a variety of data points capturing various aspects of disease. This has led most of the models to include a variety of clinical data points including disease risk factors, comorbidities, various diagnostic markers, and vital signs as inputs to the model. While this approach could potentially lead to the most accurate predictions for carefully curated test cohorts, it lacks generalization capability due to lack of veracity in the routine clinical data (Gianfrancesco et al., 2018). For example, hypertension is a clinical comorbidity believed to be correlated with unfavorable outcomes (Sheppard James et al., 2021); however, the clinical condition of hypertension, which could be either managed or unmanaged, may not be reliably captured from either medical records or direct blood pressure measurements done on a patient. In short, increase in model input data variety will certainly impact its veracity, which in turn diminishes performance and potentially decreases its generalization capacity. Furthermore, from a deployment and utility perspective, it is important to not rely upon clinical data points as they may not be easily retrievable owing to complexities in connecting to various electronic health record (EHR) systems with heterogeneous conventions in coding clinical parameters affecting their interpretations and utility in predictive models (Rajkomar et al., 2018).

We aimed to leverage a deep neural network trained model using heterogeneous cohorts of outcome matched patient data from four early epicenters of COVID-19 to predict clinical outcomes. We sought to devise the prediction system to be as practical and as easy to deploy as possible, primarily to maximize its potential impact. To this end, we set up numerous experiments to extensively study various data input contributions to the assigned surrogate outcomes and to better understand redundancies through minimizing interactions among input parameters. Finally, we aimed to create a prediction tool, which could use a parsimonious set of input data while maintaining predictive accuracy. We compared the performance of our proposed models with other approaches from the literature. To assess generalization capacity of the approach, we also further evaluated and characterized the model accuracy using a publicly available external test cohort from an independent health system, which was not utilized during the overall model training process.

## Results

**Figure 1:**
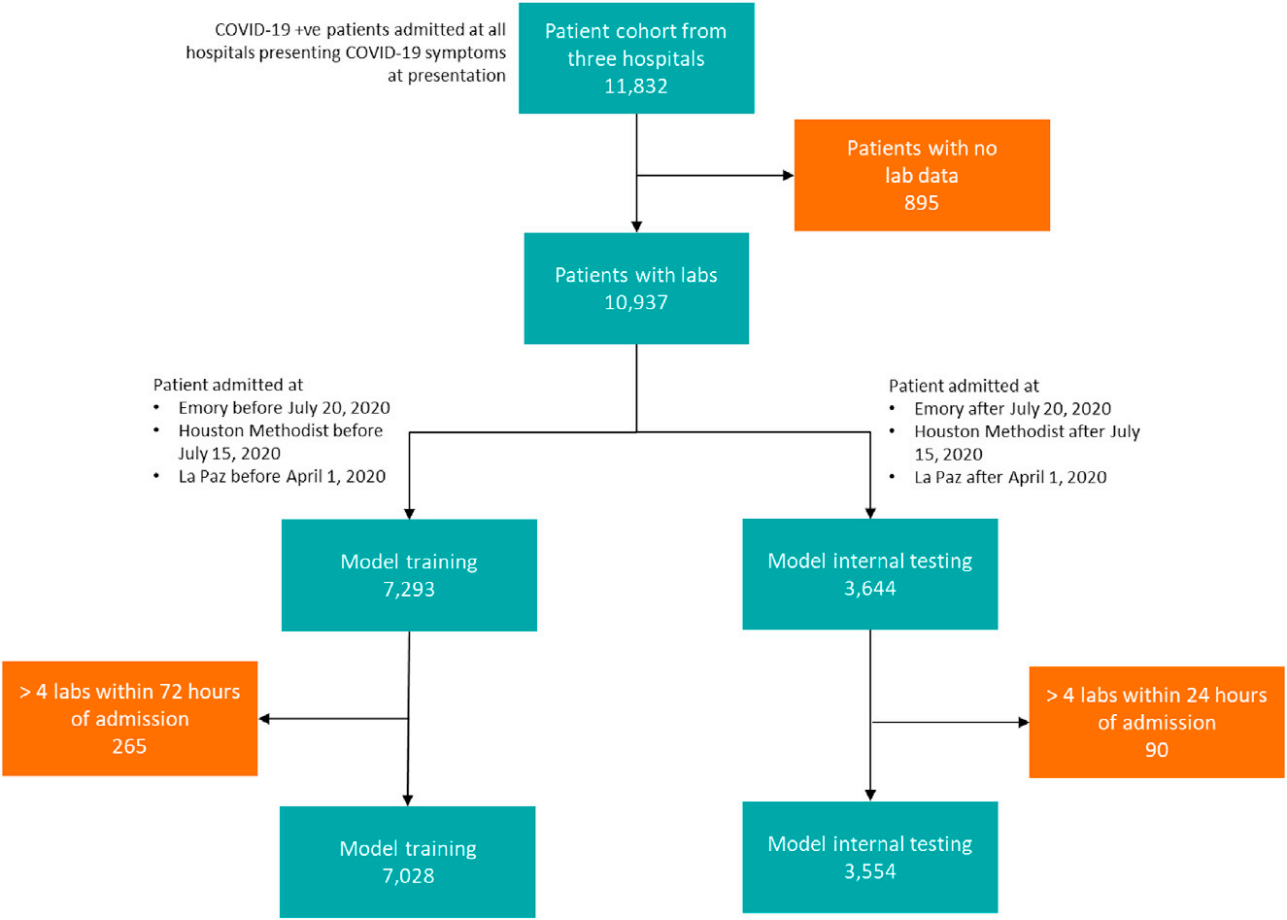
Study consort diagram Patient cohort numbers from three different sites; Emory (3114 cases), Houston Methodist (4695 cases), and La Paz (4023), used for model training, selection, and testing/validation.

**Figure 2:**
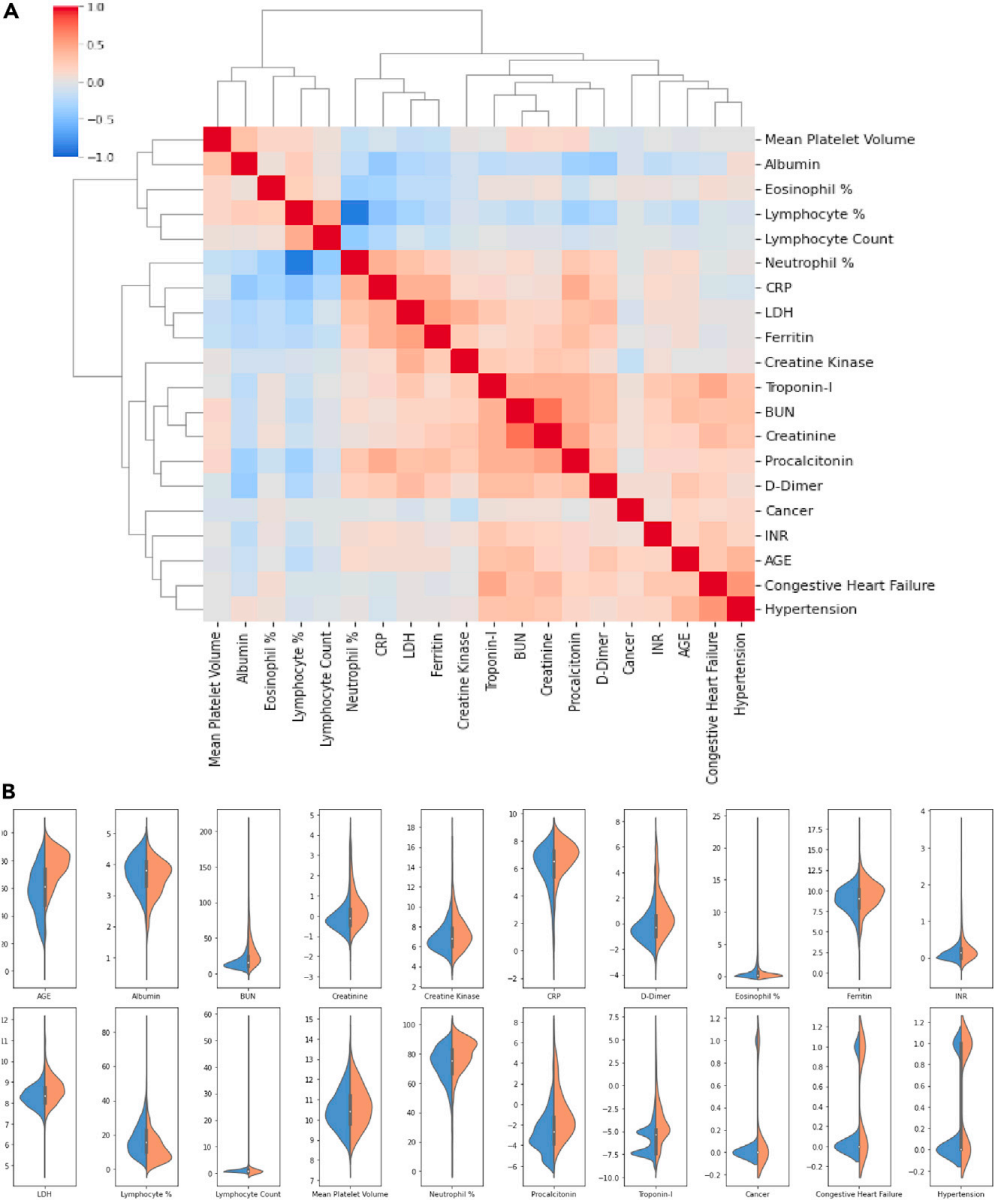
Patients' features characteristics (A) Feature correlation heatmap for top-20 variables selected by MRMR. (B) Feature distribution of corresponding variables for severity 4. For clarity, logarithmic values with base 2 are plotted for the following variables: Creatine Kinase, Creatinine, CRP, D-Dimer, Ferritin, INR, LDH, Procalcitonin, and Troponin-I.

Out of 11,832 patients from three of the institutions, 10,937 patients who met the criteria for inclusion were used to train and internal test the model. This data split into a training set and a test set consecutively based on split dates for various cohorts to maintain the ratio of 2 to 1. This was mainly to avoid any inadvertent biases and to assess performance on patient data acquired prospectively in the future. 7,293 patients (66.68%) were selected as the training set, and 3,644 patients (33.32%) were assigned to the test set. Additionally, 2,340 patients from Mount Sinai were used as an independent external testing and validation cohort. Specifically, we performed model external testing on the 739 patient cohorts admitted after April 28, 2020. The consort diagram Figure 1 depicts the cohort numbers and details from various clinical sites. Tables 1 and 3 depict a total of 57 variables comprised of laboratory tests, demographics, and comorbid conditions for patients in the training and testing (i.e., both internal and external) cohorts. Furthermore, the figure shows the statistical significance (i.e., *p value*) of the mean value difference between mortality and discharged subgroups. All the *p values* of less than 0.05 demonstrate individual potential of the variable as an independent predictor of mortality. Table 2 lists the distribution of the assigned severity levels broken down based on the specific health system. Table 2 further demonstrates both average and availability of patient data for each severity category. Figure 2 depicts both feature correlation heatmap and distributions of a set of selected features for severity level 4 (i.e., in-hospital mortality versus discharged).

**Table 1:** Baseline characteristics of the study population

Characteristics	Training cohort	Testing cohort	Mortality *p value*	External testing cohort	Mortality *p value*
No. of patients	7028	3554		739	
Age (years)	60.45 ± 0.21 (7026)	58.26 ± 0.32 (3551)	<0.0001	56.34 ± 0.85 (739)	0.2092
BMI (kg/m^2^)	31.62 ± 0.15 (3018)	31.78 ± 0.22 (1480)	0.1777	27.10 ± 0.31 (663)	0.3563
Gender: Male	2742 (5213)	1178 (2590)	0.8722	406 (739)	0.8333

**Clinical co-morbidities**					

Diabetes	1822 (5200)	875 (2580)	0.1032	165 (739)	0.9500
Cancer	254 (3163)	112 (1532)	0.0540	114 (739)	0.1421
Chronic kidney disease	997 (5199)	455 (2581)	<0.0001	94 (739)	0.0000
Chronic obstructive pulm. disease	888 (5196)	405 (2579)	0.5253	52 (739)	0.0522
Congestive heart failure	602 (3163)	281 (1532)	0.6688	88 (739)	0.0001
Hypertension	1519 (5206)	638 (2581)	<0.0001	0 (739)	

**Laboratory markers**					

Albumin (g/dL)	3.71 ± 0.01 (5902)	3.70 ± 0.01 (3068)	0.4181	3.56 ± 0.04 (402)	0.3493
BUN (mg/dL)	21.2 ± 0.22 (6554)	22.13 ± 0.36 (3207)	0.0036	27.02 ± 1.28 (363)	0.3073
Creatinine (mg/dL)	1.41 ± 0.02 (6745)	1.34 ± 0.03 (3255)	0.8921	1.58 ± 0.10 (363)	0.0011
Creatine kinase (ng/mL)	476.67 ± 151 (2236)	196.89 ± 14 (1248)	0.4687	663.12 ± 296 (24)	0.0062
CRP (mg/dL)	111.27 ± 2.11 (1797)	107.02 ± 2.81 (991)	0.0873	67.89 ± 4.72 (395)	0.0790
D-dimer (mg/L FEU)	2.61 ± 0.15 (3077)	2.80 ± 0.21 (1913)	0.7981	2.65 ± 0.19 (353)	0.9840
Eosinophil %	0.62 ± 0.02 (5886)	0.84 ± 0.03 (2811)	0.7871	(0)	
Ferritin (ng/mL)	1182.74 ± 173 (1814)	774.17 ± 74 (1622)	0.2221	771.96 ± 127 (395)	0.0000
INR	1.20 ± 0.01 (4512)	1.22 ± 0.02 (2292)	0.4918	1.27 ± 0.03 (378)	0.4882
LDH (U/L)	363.05 ± 3.32 (3334)	341.15 ± 5 (1718)	0.3875	420.53 ± 54 (323)	0.7083
Lymphocyte%	17.35 ± 0.13 (6415)	18.08 ± 0.19 (3162)	0.0709	(0)	
Lymphocyte count (k/μL)	1.10 ± 0.02 (4995)	1.38 ± 0.08 (2380)	0.7663	(0)	
Mean platelet volume	10.54 ± 0.02 (1711)	10.52 ± 0.03 (924)	0.7975	(0)	
Neutrophil %	73.74 ± 0.16 (6415)	73.01 ± 0.24 (3162)	0.0578	(0)	
Procalcitonin (ng/mL)	1.50 ± 0.22 (1602)	1.10 ± 0.22 (874)	0.8876	2.55 ± 0.94 (378)	0.7662
Troponin-I (ng/mL)	0.20 ± 0.05 (3581)	0.14 ± 0.03 (2034)	0.9128	0.55 ± 0.23 (221)	0.3549

Cohort statistics of demographic and laboratory variables are reported as mean and standard error (number of patients for which the value was recorded), and comorbidities are reported as the number of patients with comorbidity (number of patients where the value was recorded).

**Table 2:** Severity levels description, criteria, prevalence per site, and patient characteristics

	Severity 0	Severity 1	Severity 2	Severity 3	Severity 4
Description	No respiratory problem	Mild respiratory problem	Moderate to severe respiratory problem	Severe respiratory problem with organ damage	Mortality within 30 days of admission
Criteria	No O_2_ supplement required	Hypoxic patients requiring O_2_ supplement	Hypoxic patients requiring high flow nasal canula, BIPAP^a^, or invasive O_2_ supplement therapy	Same as severity 2 along with increase in SOFA^b^ score (renal, liver) by 2 and/or renal replacement therapy	In-hospital mortality within 30 days or transfer to hospice

**Prevalence per site**					

Emory hospital	838	914	377	332	318
Houston Methodist Hospital	1253	2831	126	31	454
La Paz University Hospital	1012	1142	238	107	609
Mt. Sinai Hospital (external testing)	338	1061	284	126	329

**Patient characteristic**					

Age	53 ± 0.4 (1792)	59 ± 0.3 (3357)	60 ± 0.7 (526)	65 ± 0.9 (328)	75 ± 0.4 (1023)
BMI	31 ± 0.3 (743)	32 ± 0.2 (1852)	31 ± 0.9 (93)	34 ± 2.4 (23)	29 ± 0.4 (307)
Gender (Female/Male)	580	1440	171	67	484
Diabetes	359 (1225)	981 (2783)	93 (296)	41 (104)	348 (792)
Chronic kidney disease	189 (1226)	488 (2784)	36 (295)	39 (104)	245 (790)
Hypertension	224 (1228)	681 (2786)	112 (296)	79 (104)	423 (792)
Congestive heart failure	95 (791)	334 (1943)	31 (93)	11 (23)	131 (313)
Lymphocyte (%)	21 ± 0.3 (1574)	17 ± 0.2 (3111)	15 ± 0.4 (485)	16 ± 0.6 (290)	13 ± 0.3 (955)
Eosinophil (%)	0.96 ± 0.04 (1495)	0.50 ± 0.02 (2940)	0.56 ± 0.05 (379)	0.84 ± 0.09 (207)	0.44 ± 0.03 (865)
Neutrophile (%)	69 ± 0.3 (1574)	74 ± 0.2 (3111)	77 ± 0.5 (485)	74 ± 0.8 (290)	79 ± 0.4 (955)
Creatinine	1.30 ± 0.04 (1683)	1.35 ± 0.03 (3223)	1.36 ± 0.07 (519)	1.52 ± 0.08 (321)	1.78 ± 0.06 (999)
D-Dimer	1.22 ± 0.13 (617)	1.85 ± 0.14 (1461)	3.59 ± 0.54 (350)	4.20 ± 0.86 (181)	5.45 ± 0.63 (468)
CRP	69 ± 3.9 (316)	109 ± 2.9 (918)	140 ± 6.4 (210)	116 ± 7.2 (137)	150 ± 6.7 (216)
Troponin-I	0.09 ± 0.05 (790)	0.14 ± 0.07 (1838)	0.15 ± 0.07 (291)	0.13 ± 0.04 (173)	0.64 ± 0.20 (489)
Interleukin 6^c^	85 ± 29.1 (181)	69 ± 9.6 (612)	89 ± 32.9 (166)	33 ± 9.1 (65)	169 ± 29.1 (163)

a Positive Airway Pressure.

b Sequential Organ Failure Assessment.

c IL-6 was not part of the 10-variable model owing to the low number of entries in the hospital databases.

### Feature selection results

**Figure 3:**
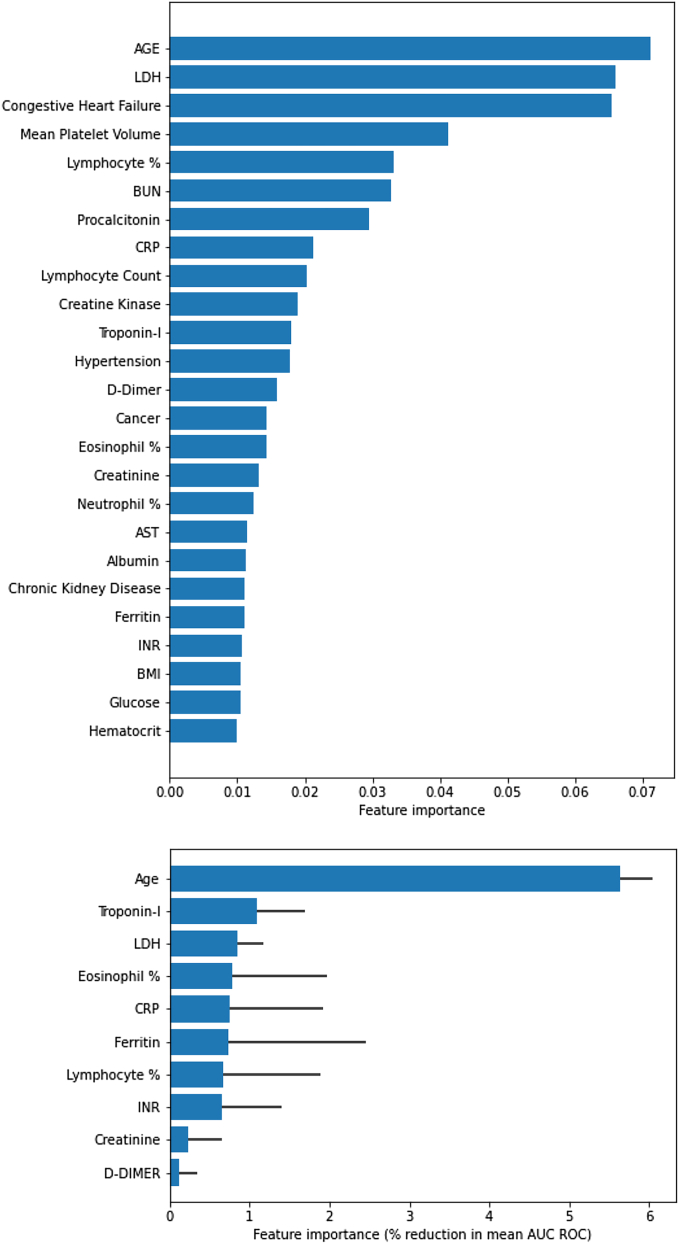
Feature importance (A) MRMR–top 20 features (B) Feature importance based on the effect on classification accuracy (i.e., decrease in AUC) using deep profiler.

We first derived ingredients of the parsimonious model out of the total of 57 features (as depicted in Tables 1 and 3) using the approach explained above. Figure 3 demonstrates the feature ranking based on the two-step approach of using MRMR, pathway-based clustering, and finally exhaustive testing of performance. Figure 3A specifically shows the ranking of 20 most discriminative features based on MRMR approach, whereas Figure 3B demonstrates the feature importance for a final selected 10 markers model.

**Table 3:** Remaining baseline characteristics of the study population

Characteristics	Training cohort	Testing cohort	Mortality *p value*	External cohort	Mortality *p value*
ALT (U/L)	42.76 ± 1.2 (6384)	40.40 ± 1.2 (3148)	0.9527	62.29 ± 16.1 (393)	0.4596
APTT	34.96 ± 0.3 (1831)	34.72 ± 0.6 (1090)	0.4485	32.26 ± 0.5 (378)	0.433
AST (U/L)	54.15 ± 2.0 (6323)	46.78 ± 1.3 (3113)	0.2604	79.76 ± 20.2 (350)	0.6108
Basophil count (k/μL)	0.02 ± 0.00 (4993)	0.02 ± 0.00 (2380)	0.7727	(0)	
Basophil %	0.30 ± 0.00 (6289)	0.30 ± 0.00 (3088)	0.8138	(0)	
Bilirubin (mg/dL)	0.62 ± 0.01 (6185)	0.65 ± 0.02 (3106)	0.0249	1.07 ± 0.11 (399)	0.7733
Chloride (mEq/L)	99.94 ± 0.1 (6756)	100.74 ± 0.1 (3258)	0.6084	102.96 ± 0.3 (405)	0.813
Eosinophil count (k/μL)	0.03 ± 0.00 (4993)	0.06 ± 0.00 (2380)	0.3004	(0)	
Fibrinogen (mg/dL)	701.86 ± 4.5 (3122)	593.81 ± 5.9 (1605)	0.6810	459.66 ± 9.4 (373)	0.0739
Glucose (mg/dL)	142.25 ± 1.0 (6786)	145.86 ± 1.6 (3276)	0.7443	140.43 ± 3.7 (405)	0.417
HCO_3_ arterial (mmol/L)	22.61 ± 0.1 (970)	22.60 ± 0.2 (478)	0.0757	22.23 ± 2.2 (19)	0.16
Hematocrit (%)	40.94 ± 0.1 (3800)	40.80 ± 0.1 (1876)	0.0016	36.76 ± 0.4 (398)	0.4439
Hemoglobin (g/dL)	13.14 ± 0.0 (6767)	13.02 ± 0.0 (3337)	0.0341	11.99 ± 0.1 (402)	0.3614
Interleukin 6 (pg/mL)	85.93 ± 9.1 (1187)	64.94 ± 9.9 (736)	0.2489	2404 ± 1211 (432)	0.7239
Monocyte%	6.96 ± 0.05 (6415)	6.86 ± 0.06 (3162)	0.9970	(0)	
Monocyte count (k/μL)	0.45 ± 0.00 (4995)	0.50 ± 0.01 (2380)	0.8253	(0)	
Neutrophil count (k/μL)	5.63 ± 0.05 (4995)	6.11 ± 0.08 (2380)	0.5532	(0)	
O_2_ saturation arterial	94.42 ± 0.2 (470)	93.73 ± 0.4 (254)	0.9082	91.98 ± 4.7 (11)	0.5197
O_2_ saturation venous	64.11 ± 1.0 (382)	61.75 ± 1.7 (152)	0.9031	64.85 ± 2.7 (64)	0.2592
PCO_2_ venous (mmHg)	43.35 ± 0.4 (431)	42.86 ± 0.7 (183)	0.6206	45.49 ± 0.6 (373)	0.1056
PCO_2_ arterial (mmHg)	36.96 ± 0.3 (1079)	36.31 ± 0.5 (508)	0.2009	37.51 ± 3.4 (18)	0.6039
Ph arterial	7.41 ± 0.00 (1011)	7.42 ± 0.00 (496)	0.3406	7.34 ± 0.03 (19)	0.1918
Ph venous	7.36 ± 0.00 (499)	7.38 ± 0.01 (195)	0.7094	7.37 ± 0.00 (373)	0.0164
Platelets (k/μL)	230.84 ± 1.2 (6763)	247.58 ± 1.8 (3335)	0.1189	(0)	
PO_2_ arterial (mmHg)	92.23 ± 1.8 (1010)	90.76 ± 2.4 (496)	0.7929	131.75 ± 26.5 (19)	0.9328
PO_2_ venous (mmHg)	40.94 ± 1.3 (478)	38.13 ± 1.5 (176)	0.6162	40.73 ± 1.6 (371)	0.9681
Potassium (mEq/L)	4.01 ± 0.01 (6694)	4.02 ± 0.01 (3227)	0.1812	4.31 ± 0.04 (358)	0.962
RBC count (m/uL)	4.45 ± 0.01 (4721)	4.42 ± 0.02 (2411)	0.9124	(0)	
Sodium (mmol/L)	136.78 ± 0.1 (6757)	137.41 ± 0.1 (3258)	0.1161	138.54 ± 0.3 (369)	0.2642
Uric acid (mg/dL)	6.77 ± 0.44 (46)	5.59 ± 0.44 (20)	0.2524	7.40 ± 1.54 (12)	
WBC count (k/μL)	7.45 ± 0.05 (6762)	8.23 ± 0.12 (3335)	0.7814	(0)	

Cohort statistics of laboratory variables are reported as mean and standard error (number of patients for whom the value was recorded).

### Parsimonious model performance

**Figure 4:**
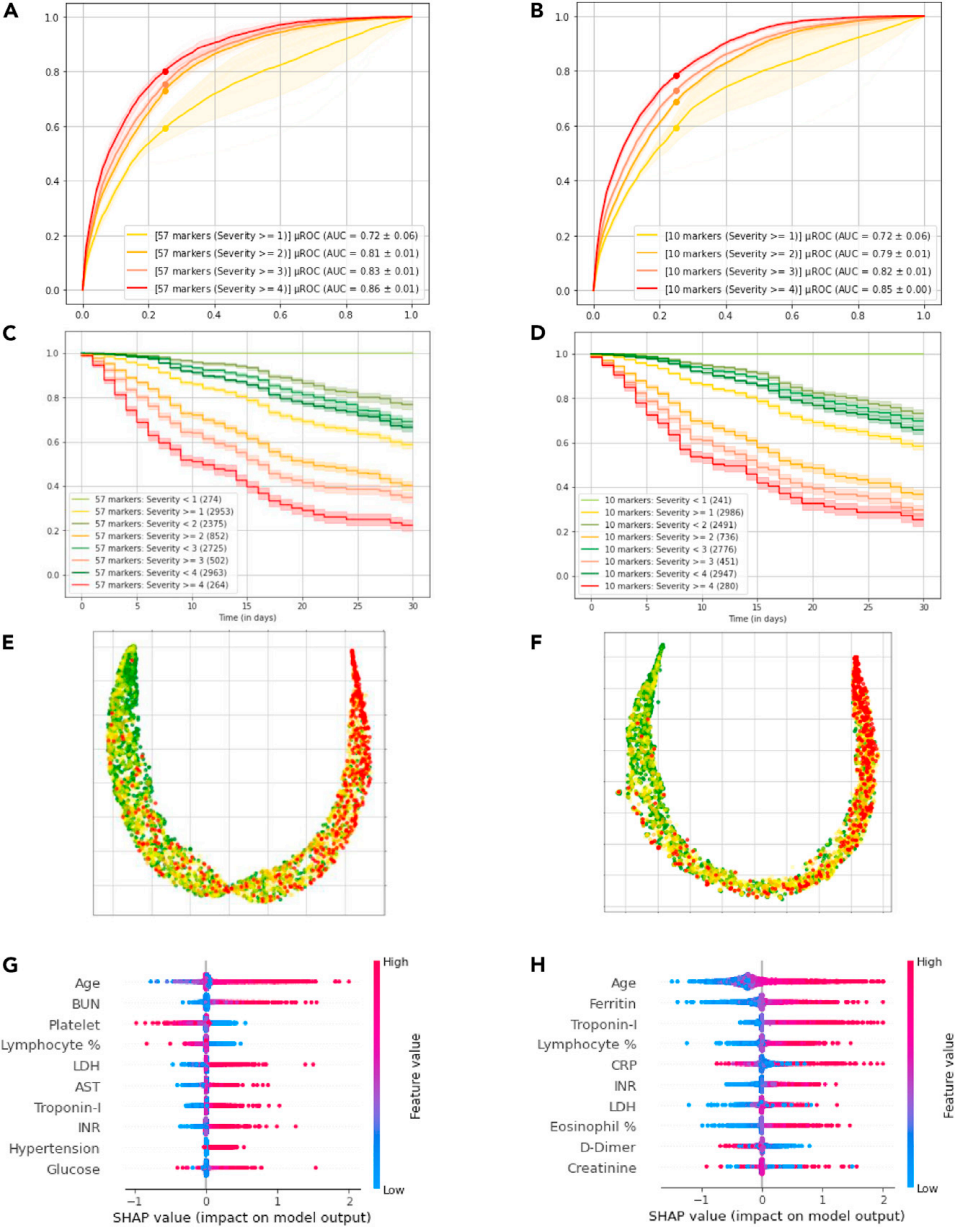
Full model (57 markers) performance versus parsimonious model (10 markers) performance (A and B) AUC curves for the predicting severity levels. (C and D) Kaplan-Meier curves comparing the shaded regions identify the 95% confidence interval. (E and F) UMAP visualization of the test dataset on the deep profiler latent space (with local neighborhood of 250) for first model in the ensemble, where green and red dots indicate severity levels 1 and 4 respectively. (G and H) SHAP summary plots of the first model in the ensemble.

We compared the performance of the deep profiler model with all the features as inputs versus the parsimonious version using only 10 markers. Figure 4 demonstrates the results for the full and parsimonious models on the left and right columns, respectively. The AUCs for the prediction of in-hospital mortality at 30 days (severity level 4) computed on the test dataset with 3,227 patient data are 0.85 + 0.01 (mean AUC + SE), and 0.85 + 0.004, for full model and parsimonious one, respectively. Similarly, the AUCs for predicting mechanical ventilation, both with and without other organ system failure, are 0.81 + 0.01 and 0.79 + 0.01. Kaplan-Meier curves for various levels of severity for the two models are also comparable. With respect to predictive accuracy of mortality (severity level = 4), the full model algorithm's positive predictive value (PPV) was 0.53 and the negative predictive value (NPV) was 0.93 (Sensitivity of 0.64 and Specificity of 0.88), whereas for the 10 markers model PPV was 0.42 and NPV was 0.94 (Sensitivity of 0.53 and Specificity of 0.85). Regarding the predictive accuracy of the severity level of 2 and above (i.e., need to use a ventilator), values for PPV and NPV were 0.50 and 0.88 (Sensitivity of 0.62 and Specificity of 0.86) for the full model, and 0.50 and 0.86 (Sensitivity of 0.61 and Specificity of 0.85), for parsimonious model, respectively.

### Model explainability

We further looked at feature distribution of various severity scores for the test cohort in the latent space (i.e., patient fingerprint as depicted in Figure 1). We observed a good separation of the classes after UMAP analysis of the latent fingerprints for both models as depicted in Figure 4, in which the sub figures (E) and (F) show the domain manifold and patients with highest and lowest severities (i.e., 4 and 1) depicted as red and green dots, respectively. Furthermore, we looked at the feature contribution for each model based on Shapley Additive Explanations (SHAP) values(Lundberg and Lee, 2017). Top ten set of most important features for both models are depicted in Figure 4. In sub figure (H), we observe that the higher values of age, troponin-I, and CRP, for example, contribute to the estimated high severities; in contrast, lower values of D-dimer have a similar effect.

### Model performance for different time intervals

**Figure 5:**
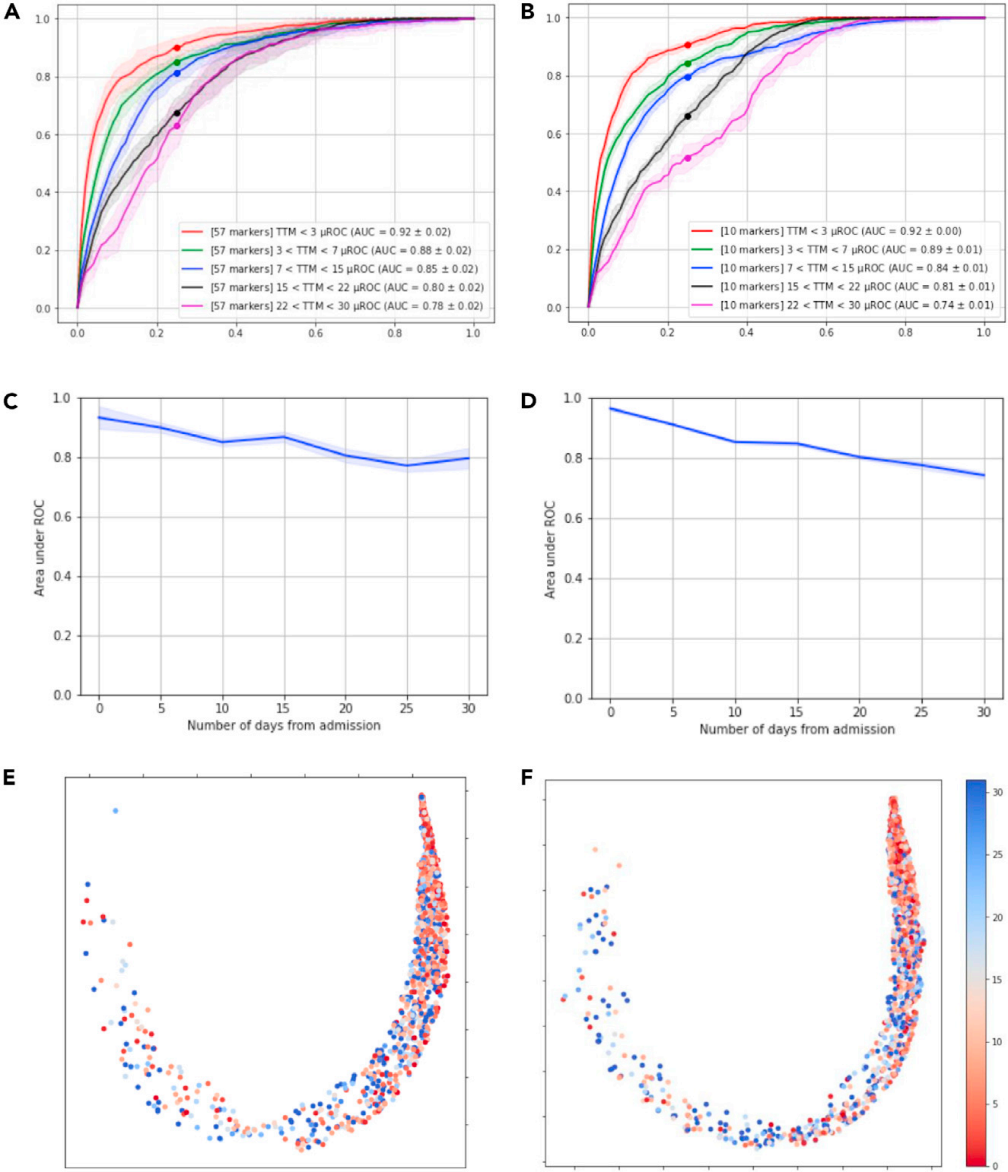
Full model (57 markers) performance versus parsimonious model (10 markers) performance for various time to event durations (A and C) show the ROC for time to mortality at 3, 7, 15, 22, and 30 days from admission for 57 markers and 10 markers model respectively. (B and D) show the progression of area under ROC for predicting severity score of 4 at 5-day intervals for 57 markers and 10 markers model respectively. Shaded regions identify the 95% confidence interval. (E and F) show UMAP visualization of time to mortality (in days) of patients with severity 4 on the deep profiler latent space (with local neighborhood of 250) for first model in the ensemble.

We also looked at model performance over time. As shown in Figure 5, the model accuracy in predicting clinical endpoints degrades as events (i.e., exacerbation to more severe conditions) happen at later days from the first encounter or admission. In other words, the prediction accuracy is higher for patients who are potentially experiencing higher levels of severity within earlier days after the initial encounter or admission. In all these comparisons, the parsimonious model has maintained the level of performance specifically in predicting mortality, whereas the full marker model has a superior performance in predicting ventilator use or predicting events happening at later timepoints. This could hint toward the importance of co-morbidities and/or redundancies in various blood various markers, which are not explicitly present in the parsimonious model.

### Model performance comparison

**Figure 6:**
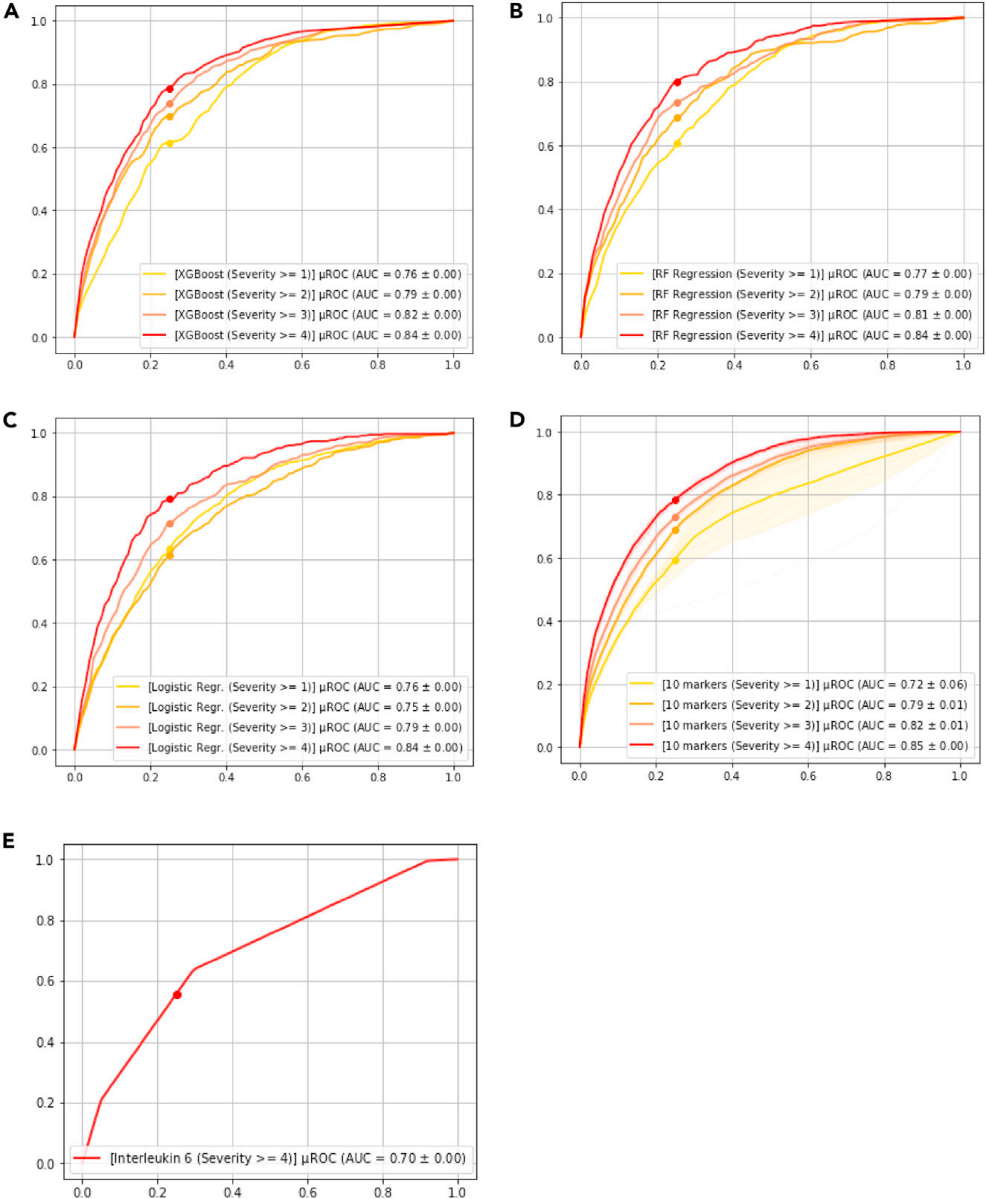
Performance of other approaches (A) XGsBoost. (B) Random Forest Regression. (C) Logistic Regression depicts performance of the machine learning models with 10 markers selected using the corresponding feature selection method. (D) Deep profiler using 10 markers. (E) Performance using Interleukin-6 as a single variate model.

To put the performance numbers in context, we also used IL-6 as a sole predictor for mortality as was suggested by McGonagle et al.(McGonagle et al., 2020), and the average AUC for the same test cohort is 0.69, which is significantly lower than both full and parsimonious models. We finally compared our approach to two popular machine learning-based approaches namely XGBoost (Chen and Guestrin, 2016) and Random Forest Regression (Couronné et al., 2018; Díaz-Uriarte and Alvarez de Andrés, 2006) and classic Logistic Regression. For XGBoost and Random Forest Regression approaches, we used grid search cross-validation to obtain the best performing model with 57 markers based on AUC and chose the top 10 performing features based on feature importance, which results in different combination of laboratory markers and comorbidities in the supplemental information Section. Figure 6 depicts the performance of the mentioned three models (i.e., XGBoost, Random Forest Regression, and Logistic Regression) in terms of AUC for predicting various levels of severity as compared to the proposed method.

### Model performance generalization

**Figure 7:**
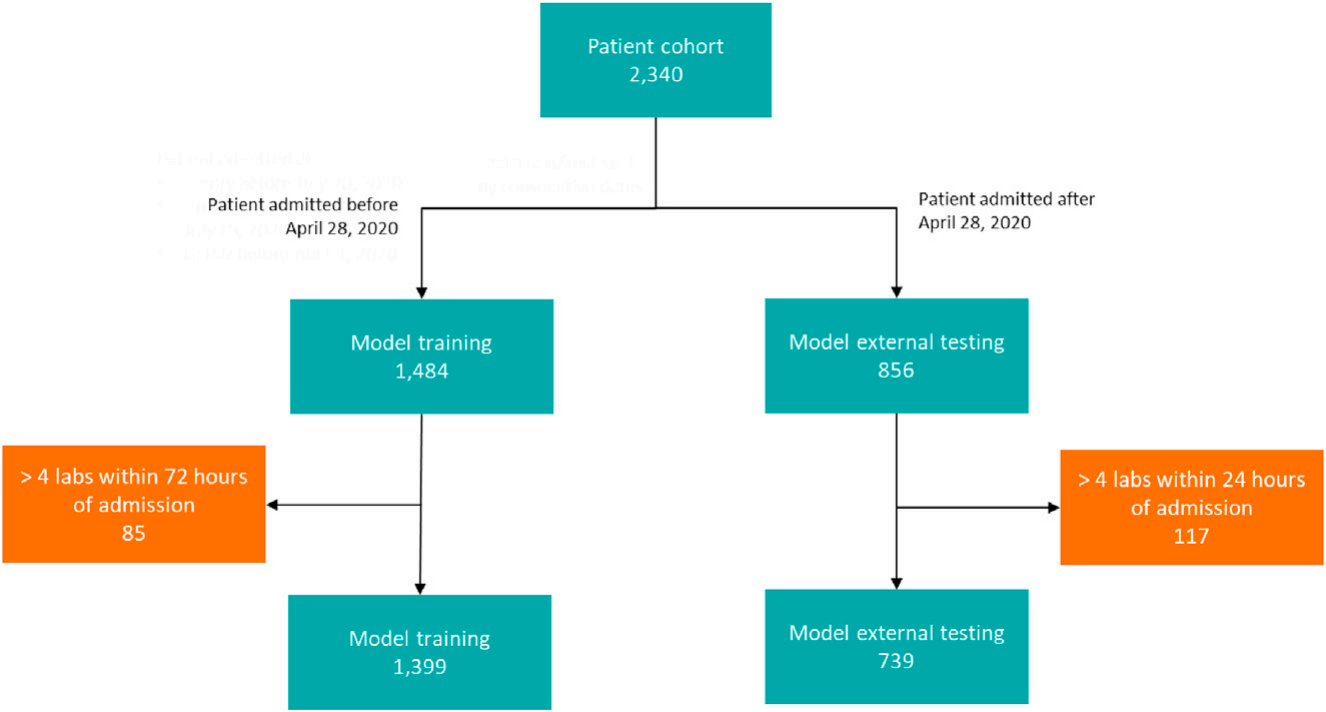
Patient cohort from publicly available data Mt. Sinai (2340), used for model external testing/validation

**Figure 8:**
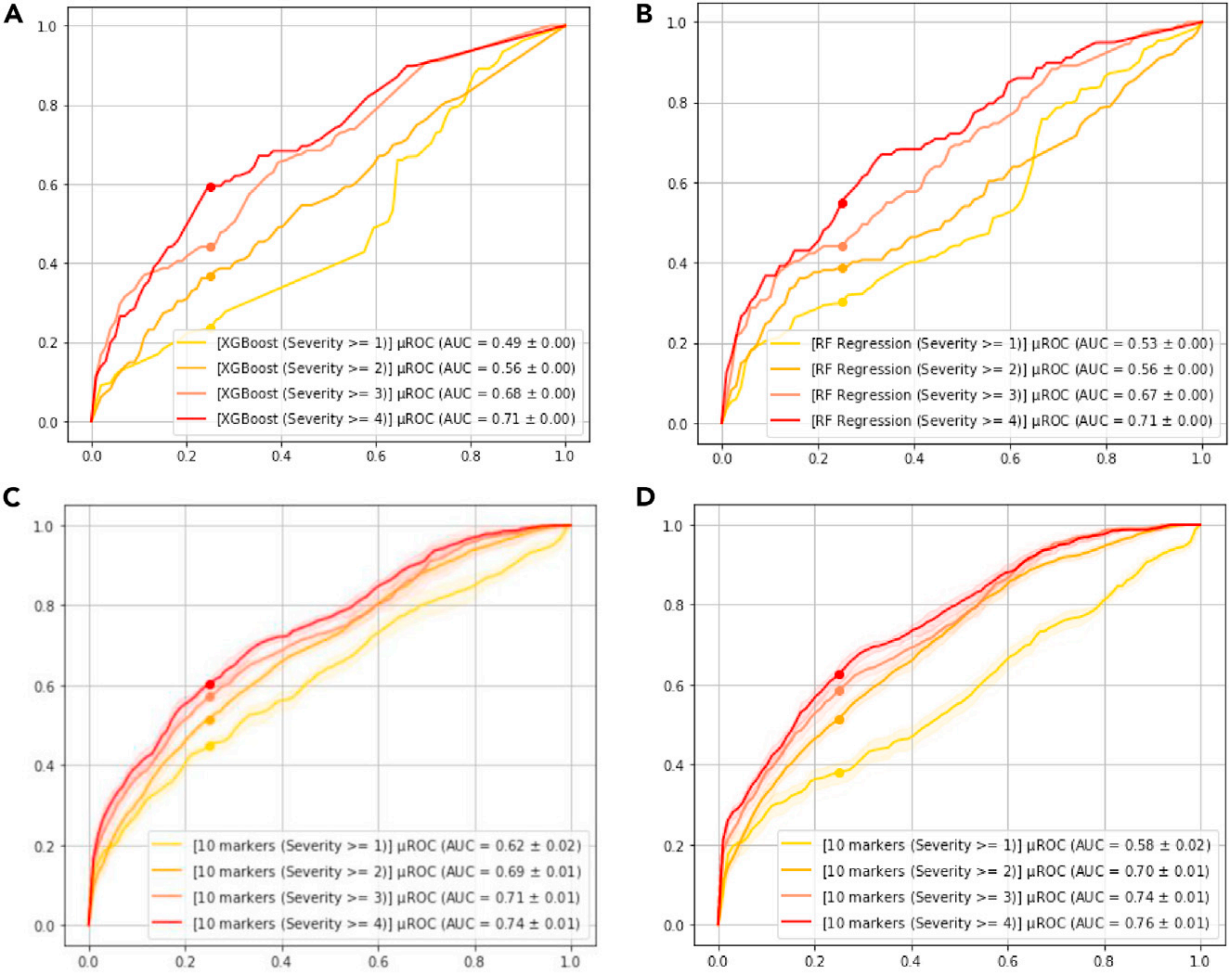
Evaluation on the external validation cohort (A) XGBoost. (B) Random Forest Regression with 10 markers selected using the corresponding feature selection method. (C) Deep profiler using 10 markers. (D) Deep profiler using 10 markers, re-trained by adding Mt Sinai training cohort to the training dataset.

To assess generalizability of model against unseen patient data from a new clinical site, we performed a series of analysis on the patient cohort from Mt. Sinai. Figure 7 depicts the consort diagram for the 2,340 records in the Mt. Sinai publicly available dataset. Specially, we used patients admitted after April 28, 2020 (i.e., denoted as “ARM1”) for the external validation. Figure 8 demonstrates the results of various models including two comparators (i.e., XGBoost and Random Forrest Regression). The 10 markers model trained using internal datasets from Emory, Houston Methodist, and La Paz outperforms other approach by a significant margin. We also further refined the 10 markers model based on the Mt. Sinai data from prior April 28, 2020; however, we did not observe any performance improvement, indicating that additional data for training did not include complementary information to what the model has been trained with.

## Discussion

In this study, we focused on developing an easy-to-use and deploy algorithm for predicting disease severity for patients with COVID-19 who were admitted to the hospital. We utilized a novel combination of feature ranking and selection methods, along with a novel deep learning-based approach to develop a 10 markers parsimonious model and algorithm from a total of 57 laboratory, clinical, and demographic variables. The training process has been done using data from 24 hospitals and three health systems in two countries.

The predicted disease severity ranges from a severity level 0 (no respiratory problem) to level 4 (in-hospital ≤30-day mortality). The prediction accuracy of severity 4 (i.e., mortality) has an AUC of 0.86 + 0.01 (mean AUC +SE) for the full model using all 57 parameters and 0.85 + 0.01 for parsimonious 10 marker model. The final selected ingredients of the 10 marker model were all shown to be independent predictors of severity in a number of prior studies (Carubbi et al., 2021; Efros et al., 2021; Fraissé et al., 2020; Long et al., 2020; Lu et al., 2021; Nie et al., 2020; Stringer et al., 2021; Wool and Miller, 2021; Wu et al., 2020; Xiang et al., 2021). We further externally validated the model using a publicly available dataset from Mt. Sinai and achieved performance with AUC of 0.74 + 0.01 for predicting mortality, which is lower than the internally validated results. We attributed this performance degradation to the lack of key blood markers namely eosinophil and lymphocyte percentages in the external validation dataset as can also be seen in the Table 3. Nevertheless, the performance of the proposed approach is shown to be superior as compared with alternative methodologies. The developed model is easy to deploy and use, primarily owing to the few numbers of required laboratory markers. It is also robust as compared to other models using clinical parameters, which may be predictive but at a wider scale are fluctuant, user-dependent, and subject to temporal influences. Our final model has nine blood biomarkers and age (Iaccarino et al., 2020), capturing various underlying biological processes also independently known to be early independent predictors of severity such as immune response (i.e., lymphocytes (Lu et al., 2021) and eosinophils (Fraissé et al., 2020)), kidney and liver functions (i.e., creatinine (Harrell et al., 1996) and LDH (Wu et al., 2020)), cardiac function (i.e., Troponin I (Efros et al., 2021; Nie et al., 2020)), inflammation processes (i.e., CRP(Stringer et al., 2021) and ferritin (Carubbi et al., 2021)), and coagulation process (i.e., D-dimer(Long et al., 2020) and INR (Wool and Miller, 2021)). Furthermore, there are studies looking at interleukins as early predictors of severity(Del Valle et al., 2020; McGonagle et al., 2020), and we have also observed their contribution; however, interleukin-6 (IL-6) specifically did not make the final cut based on the feature selection methodology we chose. Despite that, we compared the proposed model performance with an IL-6 based model as the baseline reference. Our approach as depicted in Figure 1 has many advantages, one of which is the possibility of doing further analysis on the latent feature space. This is particularly important for the model interpretations, uncertainty estimation, and to explore the relationship of unseen patient data to the patient cohort data used for training the model (Kingma and Welling, 2014). The last one is an important aspect for the clinical utility of the overall system as it could be used to create a histogram of severity distribution for similar patients within the training cohort. The similar patient severity histogram type, which could be classified as either skewed, uniform, monomodal, or random, could hint the clinical user regarding the level of uncertainty in the predicted severity level by the algorithm.

We do believe that our model has an advantage over other models as we only use the blood markers and that the physiological response to vaccination-induced immunity could potentially be expressed in these markers. A prospective validation study is warranted to further characterize the performance of the model and its clinical utility in effectively managing targeted specifically vaccinated patient population.

### Limitations of the study

Various therapeutic approaches administrated to the patients from different centers can be considered as confounding factors and can be seen as one of the limitations of this study and the resultant models. Another limitation of the study is that our data were solely gathered from unvaccinated patients. We have yet to study the accuracy of the model on vaccinated patient cohorts.

## STAR★Methods

### Key resources table

**Table:** 

REAGENT or RESOURCE	SOURCE	IDENTIFIER
**Deposited data**		

SDY1662 - an inflammatory cytokine signature predicts Covid-19 severity and Survival	Mount Sinai Health System	https://www.immport.org/shared/study/SDY1662

**Software and algorithms**		

Atellica®COVID-19 severity algorithm App	Siemens Healthineers	https://atellica-covidalgo.azureedge.net/

### Resource availability

#### Lead contact

Further information and request for resources should be directed to the lead contact, Ali Kamen (ali.kamen@siemens-healthineers.com).

#### Materials availability

This study did not generate any new reagents.

### Experimental model and subject details

Our study does not use experimental models typical in life sciences.

### Method details

#### Cohort characteristics

We obtained deidentified retrospective records from 14,172 patients admitted due to a confirmed SARS-CoV-2 infection from La Paz University Hospital (La Paz) in Madrid, Spain (3 hospitals), Emory Healthcare System (Emory) in Atlanta, Georgia (6 hospitals), Houston Methodist Hospital System (Houston Methodist) in Houston, Texas (7 hospitals) and Mount Sinai Health System (Mount Sinai) in New York, New York (8 hospitals). De-identification was done in accordance with the USA Health Insurance Portability and Accountability Act (HIPAA) and the European General Data Protection Regulation (GDPR). Informed consent waivers were approved by the institutional review boards or ethics committees for La Paz, Emory, and Houston Methodist. External and independent data from Mount Sinai were publicly available and obtained via IMMPORT shared data repository (see https://www.immport.org/). Patients from Emory and Houston Methodist were admitted between March 1, 2020 and August 8, 2020, and patients from La Paz were admitted between February 24, 2020 and May 19, 2020. The data from Mount Sinai included patients admitted between March 21, 2020 and June 23, 2020. The records included demographic information, clinical conditions, comorbidities (e.g., chronic kidney disease), and laboratory parameters (e.g., lactate dehydrogenase) taken at various timepoints after the first encounter, and various diagnostic tests (e.g., SpO_2_) obtained during the patient's initial encounter up to 72 hours following evaluation. Tables 1 and 3 summarizes the cohort characteristics and detailed epidemiological, demographic, clinical, and laboratory data. We reserved the publicly available Mount Sinai dataset for external testing and did not use it during model training and selection.

#### Clinical outcome based severity score assignment

For all these patients, we assigned a severity score to better characterize clinical endpoints as defined by Table 2. The severity scores are assigned based on the worst condition of the patient during the course of the hospital stay on an ordinal scale from 0 to 4. The definitions of the various scales were primarily based on Berlin criteria and SOFA score (Ferguson et al., 2012; Lambden et al., 2019), however, they were modified to align better with COVID-19 disease characteristics and to have a harmonized approach based on data availability from the different sites used for this study. Based on our defined severity scale, a severity score of 0 indicates no respiratory problem, whereas severity levels 1 and 2 are reserved for patients with mild/moderate and severe respiratory problems, respectively. A severity score of 3 is assigned to patients, who had a severe respiratory problem along with other organ system failures with a focus on liver and kidney during the hospital stay. Finally, severity level 4 denotes either in-hospital mortality within 30 days or transfer to hospice.

#### Deep profiler prediction algorithm

The schema of the proposed (i.e., deep profiler) method, which is based on deriving a patient fingerprint from various demographic, clinical, and laboratory parameters and using it to predict severity score is shown in the below Figure. Deep profiler consists of three main parts (i.e., networks): an encoder network for extracting prominent features represented in a latent space, which is also referred to as the patient fingerprint, a decoder network for reconstructing the input data to ensure data fidelity of the latent feature representation, and finally a severity classifier network, which is trained to estimate the severity score (Lou et al., 2019). We chose a variationalautoencoder (VAE) based approach to provide a probabilistic representation of input parameters gathered at the first clinical presentation in a latent space (Kingma and Welling, 2014). In this approach, the encoder output is a probability distribution for each latent attribute corresponding to an input instance. The encoder consists of four fully connected layers with 64, 32, 32 and 16 channels respectively. Each fully connected layer is followed by a batch normalization layer and a leaky rectified linear activation operation (leaky ReLU) with slope of 0·2. The decoder consists of four fully connected layers with 16, 32, 32 and 64 channels respectively, with each fully connected layer followed by a batch normalization layer and a rectified linear (ReLU) activation operation. The advantage of VAE is twofold. First, the entire autoencoder set of networks (i.e., encoder and decoder) could be trained with entire patient data in an unsupervised manner without the knowledge of their specific clinical outcome and labels. This is specifically important for the problem at hand, since the complex interactions among input datapoints could be disentangled using a potentially much larger set of data points. Secondly, variation aspect of autoencoder forces the latent state representation to be smoother as compared to standard autoencoders and that helps with the improved generalization capacity. We have further outlined advantages of VAE in the supplemental information section, which also include more principled way of dealing with missing input point values, by learning a disentangled representation in the latent space.

**Figure:**
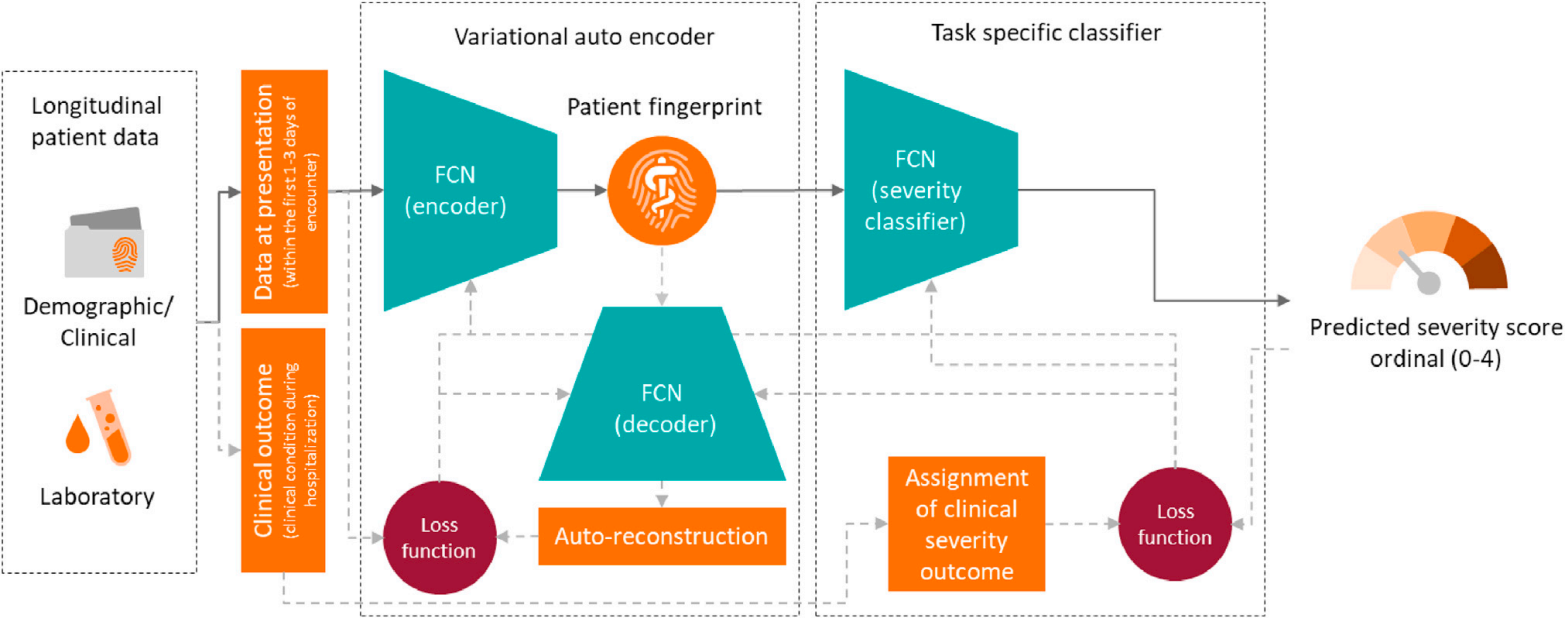
Schematic of the deep profiler approach during training and inference phases based on three fully connected networks (FCN) The solid arrows show the data flow during the inference or testing, whereas dashed gray arrows show additional data flow required during the training of the networks (i.e., encoder, decoder, and severity classifier).

We employ a classifier network to predict the severity score based on the latent fingerprint variables. Since the order among various severity levels is important, we formulated severity score prediction problem as an ordinal regression one. We first train the weights of the neural network on the ordinal classification task to predict severity levels, and subsequently use an ordinal linear regressor to obtain severity scores between 0 and 4. The ordinal classification task over 5 values is reformulated as 4 binary classification tasks (Gutiérrez et al., 2016). Thus, the severity prediction classifier network outputs 4 binary outputs, corresponding to whether severity level is greater or equal to 1, 2, 3 or 4 respectively. The network consists of 4 fully connected layers. The first 3 fully connected layers have 32 channels are followed by a rectified linear activation. The fourth fully connected layer maps the 32-channel feature to 4 output variables with sigmoid activation. To avoid overfitting, a dropout of 0·25 is employed after the first and second fully connected layers. The network parameters are optimized by minimizing the combined loss of variational auto-encoder with L^1^ reconstruction loss and the binary cross entropy loss on the output of the severity classifier using Adam with a learning rate of $3\times 10^{-4}$. Our model implementation is based on PyTorch (http://pytorch.org).

Given the output scores of the 4 binary network classifiers, we employ an ordinal L^2^-regularized linear regressor (Rennie and Srebro, 2005) to obtain a severity score between 0 and 4. We use the All-Threshold based construction as described in the paper by Rennie et al.(Rennie and Srebro, 2005), which bounds the mean absolute error between the true and predicted severity levels. The parameters of ordinal linear regressor were obtained by performing grid search with 5-fold cross validation using scikit learn, a machine learning library in Python (https://scikit-learn.org/).

#### Model calibration and clinical event likelihood computation

Deep profiler outputs the severity risk score between 0-4 for a patient, given the input parameters at the time of admission. The concordance index (CI) (Couronné et al., 2018) of the predicted severity levels, which quantifies the quality of rankings, on the internal testing dataset and external validation dataset of Mt Sinai was 0.71 and 0.64, respectively. In addition to the risk score, the outputs of the severity classification network can also be used to compute the likelihood of the clinical events of acute respiratory failure, end organ damage with respiratory failure and mortality, corresponding to severity levels ≥2, ≥3 and 4 respectively. However, the softmax output of the deep network classification networks is often not well calibrated to be interpretated as likelihoods. To address this, we fit a logistic regression model to the outputs of the severity classification network. We repeat this process for each deep profiler in the ensemble, then use the mean likelihood of the ensemble as the overall likelihood for the clinical event.

#### Data preprocessing

The laboratory measurements recorded at the time of admission depends on several factors such as the patient's symptoms and thus, in retrospective data cohorts, all laboratory measurements are not available for all the patients. Furthermore, other clinical information such as comorbidities may not be available. Table 1 shows the number of patients for each characteristic. We preprocess the lab measurements to normalize the input data distributions. For certain laboratory measurements where the input distribution is heavy tailed, we transform them to logarithmic space. All variables are centered and scaled based on median and interquartile range for increased robustness to outliers. All the missing variables are imputed using the median value.

#### Model training

Training deep profiler to minimize the combined loss of the variational auto encoder as well as ordinal regression posits several challenges. For instance, uneven data and outcome distributions as reported in Table 2, e.g., the number of patients for severity level 2 is significantly more than severity level 4. This is addressed by appropriately weighting the loss terms. The losses corresponding to 4 binary classifiers are weighted as {0·3, 1, 1·5, 4} respectively based on distribution of samples over different outcomes and relative importance of the task. Another challenge is that classification loss can significantly bias the encoder weights in the initial epochs, making it difficult to learn a smooth manifold. This is addressed by only optimizing the parameters of the variational auto-encoder for the first 10 epochs and optimizing all the networks jointly for the subsequent epochs. all the networks are optimized jointly.

While use of variational auto-encoder helps in dealing with model bias due to missing data via implicit imputation, there is still a risk that a single deep profiler model trained over entire training set can still incorporate such a bias. To further mitigate this risk, we train an ensemble of deep profilers over 10 different splits of the training dataset. Each split partitions the training dataset into a parameter training set and model selection set with 9 to 1 ratio, while maintaining the original ratio of samples for each severity level. Parameter training set is used for learning the weights via backpropagation and the model selection set is used to select the best model over epochs. Given the ensemble of 10 deep profilers, the overall severity score is computed as the average of the severity scores. In addition to the overall severity score, we also report the 95% confidence interval.

#### Missing values

Missing input data is a prevalent problem specifically for larger cohorts of patients in which consistent data acquisition and availability is hard to ensure. This posits a greater challenge to the acceptance of machine learning based prediction models, since missingness of data can be attributed to the level of severity of the patient (fewer tests may have been done since the patients didn't exhibit severe symptoms) and machine learning models are potential learn the pattern of missingness as a predictive feature.

There are different approaches to deal with missing input data points, and these range from leaving the entire record with missing data point aside, to performing missing data imputation as part of pre-processing step (e.g., using K-nearest neighbor method(Faisal and Tutz, 2017), singular value decomposition(Troyanskaya et al., 2001), or variational auto-encoders(Qiu et al., 2020)), or most preferably using implicit data imputation within the prediction task. In this work, since our prediction network has a variational auto-encoder embedded, we use a hybrid approach, which consists of three steps. First, for all missing data points for each patient record, we perform median based imputation. Pseudocode for pre-processing is detailed in the Algorithm S1. Second, during the training of the VAE, we randomly drop various data points from each patient record (de Jong et al., 2019). In this scenario, the auto-reconstruction loss is computed only on non-missing values. In this case, the latent space representation (i.e., patient fingerprint) becomes inherently robust with respect to missing variables with different patterns of missing at random(Phung et al., 2019). Pseudocodes for training procedure and specifically the training loss are detailed in the Algorithms S2 and S3, respectively. Finally, VAE inherently learns the distribution of the input data by estimating mean and standard deviation of the mapped latent parameters assuming a mixture of Gaussian distributions. Any missing data points at the input could potentially be less severely affecting the latent parameters and the overall distribution formed by the entire training cohort. Finally, since we train an ensemble of networks trained on various folds with random drop-out patterns, we further ensure that the effect of data missingness is minimized. In order to assess the effectiveness of these choices, we conducted additional analysis to study the missing data distribution. Figure S1A shows the histogram of the patients with different number of missing input parameters. The pseudocode for the inference used during evaluation is brought in the Algorithm S4. Nearly half of the patient population has 50% of more of the 57 input parameters missing, thus, the risk of missing data to be a feature is certainly a possibility. We then set up an experiment, where we used the number of missing patient parameters as a feature and studied the patient distribution for each severity level. Figure S1B shows the violin plots for each severity level; notice that the violin plots for each severity level are quite similar suggesting the number of missing data itself isn't a strong indicator of severity in this dataset.

We also evaluated the performance of deep profiler on the subset of data where all the 10 markers were obtained at the time of admission. Figure S2 shows the AUCs as well as the UMAP projection of the subset of the data on the training manifold. While this subset of data only has 300 patients, we observed a marginal increase in performance among patients with higher severity while a drop in performance among patients with lowest severity. More importantly notice that the patient samples are projected uniformly over the manifold, thus suggesting the model is not implicitly grouping patients with different number of missing parameters at different location on the manifold.

#### Parsimonious model creation

To improve the overall model performance and to increase the utility of the overall predictive system, it is important to devise a parsimonious model with as few input parameters as possible. An exhaustive optimal feature selection algorithm is a non-deterministic polynomial-time (NP)-hard problem as the number of combinations changes based on the factorial of the number of features. Aside from unsupervised feature selection techniques in which no target label is considered and the goal is to reduce the number of redundant variables through a correlation analysis, we have several categories of supervised approaches. The three categories are filter-based, wrapper-based and intrinsic approaches (Saeys et al., 2007). Filter-based methods are based on conventional statistical approaches and for the most part do not consider complex non-linear relationship among the features and target labels. Wrapper-based methods are primarily based on iterative selection and/or elimination of features and qualification of the final selected set based on the algorithm performance to predict the target. These methods which are essentially search based are usually computationally expensive and require model training and testing for the number of selected feature set candidates. Finally, in embedded methods, the feature selection is formulated as an explicit part of the predictor loss function in addition to the prediction error. This could also be thought of enforcing regularization on the decision function or the decision boundary (Muthukrishnan and Rohini, 2016). We used a modified two step feature selection method (Drotár and Gazda, 2016; Wang et al., 2007). The modification is primarily based on bringing in the knowledge of the relationship among various blood biomarkers and clinical co-morbidities. For each of the laboratory markers, we assigned them to clusters based on the relationship with the following underlying physiological processes such as immune response, inflammation process, coagulation pathways, cardiac function, and liver and kidney functions. We used minimum redundance maximum relevance (MRMR) technique to rank order features based on the importance(Mandal and Mukhopadhyay, 2013) and reduce the number of candidates to 20. Furthermore, we chose less than 100 combinations of markers, taking in into account equitable representations from various clusters as stated above. For all these combinations, we trained and evaluated the deep profiler performance on the test dataset and chose the winning combination with the highest performance on basis of the area under the receiver-operator characteristic (ROC) curve (i.e., referred to as AUC).

#### Description of baseline approaches

We compared the performance of proposed approach with three approaches, namely, XGBoost^49^, Random Forest Regression^50^ and Logistic Regression. In order to have a fair comparison, we used a feature importance approach for each specific method to select the top ten most prominent markers for model creation(Díaz-Uriarte and Alvarez de Andrés, 2006; Genuer et al., 2010).

Specifically, for XGBoost, we utilized a hyperparameter search with 5-fold cross validation on the training set to identify the best performing model with all input parameters that minimizes logistic regression loss function and achieves the highest area under the curve. The search was conducted using XGBoost python library (https://xgboost.readthedocs.io/), with following parameter ranges: max_depth ϵ {2, 3, 4, 5}, min_child_weight ϵ{1, 2, 3, 4, 5}, subsample ϵ {0.5, 0.6, 0.7, 0.8, 0.9, 1.0}, colsample ϵ {0.7, 0.8, 0.9, 1.0}, with the rest of the parameters set to default. We then used the relative feature importance of the input parameters from the best performing model to select the top 10 performing features. The selected features and their feature importance are shown in Figure S3A. We then subsequently train the parsimonious XGBoost model with the selected 10 features over the training set and report the results for comparison over the internal testing and external validation datasets.

For Random Forest Regression, we followed a similar feature selection and model optimization pipeline as described for XGBoost. The hyperparameter search for the model with all markers was done using scikit learn, a machine learning library in Python (https://scikit-learn.org/) with following parameter ranges: n_estimators ε {100, 200, 300}, max_features ε {'auto', 'sqrt'}. The selected 10 features with corresponding feature importance are shown in Figure S3B.

Similarly, for Logistic Regression, we used the 10 features selected using the Random Forest model, as it marginally outperformed the XGBoost model. We then subsequently trained the logistic regression model using 5-fold cross validation, while searching over the following hyperparameters: C ϵ {10^-2^, 10^-1^, 1, 10, 10^2^}, solver ϵ {liblinear, lbfgs}, with class_weight set to “balanced”.

Finally, for the proposed deep profiler approach, we used the hybrid feature selection as explained in the STAR Methods section.

#### Generalization

Our datasets used for training, model selection, and testing are from three hospital systems, namely Emory, Houston Methodist, and La Paz. To assess the model generalizability, we used an external publicly available dataset from Mount Sinai Hospital. All testing on the Mount Sinai data was performed using the portion of the dataset which was taken after April 28, 2020. Furthermore, to assess the upper bound of performance, we trained a model which included all the data from the three internal hospital systems along with the portion of the data from Mt. Sinai taken prior to April 28, 2020. We followed the same training procedure as described before, except that we initialized the model parameters with the learned parameters obtained from training only on the data from three internal hospital systems. We have summarized the results in the main manuscript Figure 8. Aside from the proposed algorithm results on the external validation data, Figure 8 in the main manuscript also include the results from other approaches namely XGBoost and Random Forest Regression. We observed a moderate degradation of performance for example for mortality prediction (i.e., severity 4) from AUC of 0·85 to 0·74, however, we attribute this to missingness of the while blood cell counts in for the entire external validation cohort. As we have seen in the main manuscript Figure 4 for example, Lymphocyte percentage is predictive of the clinical outcomes and its missingness will most likely adversely affect the performance.

#### Deployment/Dissemination

The final parsimonious model which has nine laboratory markers and age is available using the following URL: https://atellica-covidalgo.azureedge.net/. The application computes the estimated severity score and three likelihoods in percentages for various severe clinical events. The likelihoods are computed based on calibrated ensemble output of ten deep profiler networks as explained earlier in the text.

### Quantification and statistical analysis

#### Model performance evaluation

The predictive performance of a model was evaluated on a testing data cohort of 3,554 patients (see Figure 1) by reporting the AUC to assess discriminative ability for each of the severity levels, the Kaplan-Meier survival plots to analyze time to events (mortality, ventilator use) as well as other evaluation metrics including positive predictive value (PPV), negative predictive value (NPV), sensitivity and specificity. For AUC results, we also considered splitting the clinical outcomes to various time intervals after the initial presentation of the disease to capture possible performance differences for clinical outcomes based on their time elapsed from the first encounter. We used the Uniform Manifold Approximation and Projection (UMAP) method (McInnes et al., 2020) to display the latent fingerprint distribution of the data. Using UMAP, we projected the 8-dimensional latent fingerprints to 2-dimensional points such that similar input data are closer and dissimilar points are farther apart with high probability. The UMAP plot shows various instance of predicted severity levels in relation to one another.

## Data Availability

Data: The data includes patient information and hence cannot be made publicly available. Any additional information required to reanalyze the data reported in this paper is available from the lead contact upon request. Software: The software developed in this work, AtellicaCOVID-19 Severity Algorithm App, is available online via a public webpage (https://www.immport.org/shared/study/SDY1662). Code: The pseudo codes of the machine learning algorithm and data pre-processing are included as supplement items (Algorithms S1–S4).

## Supplemental information

Document S1. Figures S1–S3 and Algorithms S1–S4

## Footnotes

Supplemental information can be found online at https://doi.org/10.1016/j.isci.2021.103523.
