# Supplementary material for: A deep learning approach for predicting severity of COVID-19 patients using a parsimonious set of laboratory markers
Source: iScience. 2021 Nov 27;24(12):103523. doi: 10.1016/j.isci.2021.103523 (PMC8626152; doi:10.1016/j.isci.2021.103523)
Supplement: Document S1. Figures S1–S3 and Algorithms S1–S4 [file mmc1.pdf]

iScience, Volume *24*

Supplemental information

A deep learning approach for predicting severity of COVID-19 patients using a parsimonious set of laboratory markers

Vivek Singh, Rishikesan Kamaleswaran, Donald Chalfin, Antonio Buño-Soto, Janika San Roman, Edith Rojas-Kenney, Ross Molinaro, Sabine von Sengbusch, Parsa Hodjat, Dorin Comaniciu, and Ali Kamen

SUPPLEMENTAL MATERIAL

Algorithm 1: Pseudo code for the preprocessing the data.

Input

$\mathcal{D}_{demo}$ patient demographic information
$\mathcal{D}_{labs}$ clinical laboratory measurements
$\mathcal{D}_{comorbs}$ co-morbidity information

Output

$\mathcal{D}_{all}$ combined measurements

procedure PREPROCESS
    $mask \leftarrow$ All patients with number of available laboraoty markers < 4
    $\mathcal{D}_{demo} \leftarrow \mathcal{D}_{demo}[mask]$
    $\mathcal{D}_{labs} \leftarrow \mathcal{D}_{labs}[mask]$
    $\mathcal{D}_{comorbs} \leftarrow \mathcal{D}_{comorbs}[mask]$
    $labs = \{$ALT, APTT, AST, Creatinine, CRP, D Dimer, Ferritin, Fibrinogen, Hematocrit, INR, LDH, Procalcitonin, Troponin-I, Creatine Kinase, Bilirubin$\}$
    for $lab \in labs$ **do**
        $\mathcal{D}_{labs}[all, lab] = log_2(\mathcal{D}_{labs}[all, lab])$
    $\mathcal{D}_{all} \leftarrow concatenate(\mathcal{D}_{demo}, \mathcal{D}_{labs}, \mathcal{D}_{comorbs})$
    for $f \in All features in \mathcal{D}_{all}$ **do**
        $m \leftarrow median(\mathcal{D}_{all}[all, f])$
        $s \leftarrow interquartile\_range(\mathcal{D}_{all}[all, f])$
        $\mathcal{D}_{labs}[all, f] = \frac{(\mathcal{D}_{labs}[all, f] - m)}{s}$
    return $\mathcal{D}_{all}$

Algorithm 2: Pseudo code for the training algorithm.

Input

- $\mathcal{D}_{demo}$ patient demographic information
- $\mathcal{D}_{labs}$ clinical laboratory measurements
- $\mathcal{D}_{comorbs}$ co-morbidity information
- $\mathcal{D}_{severity}$ severity level

Output

- $\mathcal{M}_{best}$ Deep Profiler model

procedure TRAIN
    $epoch \leftarrow 0$
    $\mathcal{D}_{all}, \mathcal{D}_{mask} \leftarrow$ PREPROCESS($\mathcal{D}_{demo}, \mathcal{D}_{labs}, \mathcal{D}_{comorbs}$) ▷ Pre-process patient information into one container
    $train, test \leftarrow$ SPLIT($\mathcal{D}_{all}, \mathcal{D}_{severity}, 9$)

    while $epoch \leq MaxEpochs$ **do**
        $\mathcal{O}_{labels}, \mathcal{O}_{recon}, \mathcal{O}_{mean}, \mathcal{O}_{var} \leftarrow$ DEEPPROFILER($\mathcal{M}, \mathcal{D}_{all}[train]$)
        $\mathcal{L}_{mse}, \mathcal{L}_{KL} \leftarrow$ RECONSTRUCTIONLOSS($\mathcal{O}_{recon}, \mathcal{O}_{mean}, \mathcal{O}_{var}, \mathcal{D}_{all}[train], \mathcal{D}_{mask}[train]$)
        $\mathcal{L} \leftarrow \mathcal{L}_{mae} + \beta \cdot \mathcal{L}_{KL}$
        if $epoch > LabelTrainingEpoch$ **then**
            $\mathcal{L}_{severity} \leftarrow$ LABELINGLOSS($\mathcal{O}_{labels}, \mathcal{D}_{severity}[train]$)
            $\mathcal{L} \leftarrow \mathcal{L} + \lambda \cdot \mathcal{L}_{severity}$
        $\Delta\mathcal{M} \leftarrow \nabla_{\mathcal{M}}\mathcal{L}$ ▷ Use backpropagation to compute the gradient
        $\mathcal{M} \leftarrow \mathcal{M} + \alpha \cdot \Delta\mathcal{M}$ ▷ Update the model parameters

        if MODELSCORE($\mathcal{M}, \mathcal{D}_{all}[test], \mathcal{D}_{severity}[test]$) $>$ MODELSCORE($\mathcal{M}_{best}, \mathcal{D}_{all}[test], \mathcal{D}_{severity}[test]$) **then**
            $\mathcal{M}_{best} \leftarrow \mathcal{M}$

        $epoch \leftarrow epoch + 1$
    return $\mathcal{M}_{best}$

Algorithm 3: Pseudo code for computing the training loss function.

Input

- $\mathcal{O}_{recon}$ reconstructed data obtained using deep profiler
- $\mathcal{O}_{mean}$ patient latent vectors obtained using deep profiler
- $\mathcal{O}_{var}$ logarithmic of patient latent vector variances obtained using deep profiler
- $\mathcal{D}_{all}$ pre-processed patient data
- $\mathcal{D}_{mask}$ indicator of missing patient measurements

Output

- $\mathcal{L}_{mae}$ reconstruction loss
- $\mathcal{L}_{KL}$ regularization loss

procedure ReconstructionLoss
    $N \leftarrow length(\mathcal{D}_{all})$
    for $i \in \mathcal{D}_{mask}$ **do** ▷ Obtain a indicator array of available measurements
        $mask[i, j] \leftarrow$ *0* **if** $\mathcal{D}_{mask}[patient = i, measurement = j]$ *is null* **else** *1*
    $\mathcal{L}_{mae} \leftarrow \frac{1}{N} \sum |\mathcal{O}_{recon}[mask] - \mathcal{D}_{all}[mask]|$
    $\mathcal{L}_{KL} \leftarrow -0.5 \cdot \frac{1}{N} \sum \left(1 + \mathcal{O}_{var} - ||\mathcal{O}_{mean}||^2 - \exp\left(\mathcal{O}_{var}\right)\right)$
    return $\mathcal{L}_{mae}, \mathcal{L}_{KL}$

Input

- $\mathcal{D}_{demo}$ patient demographic information
- $\mathcal{D}_{labs}$ clinical laboratory measurements
- $\mathcal{D}_{comorbs}$ co-morbidity information

Output

- $\mathcal{D}_{all}$ combined measurements

procedure LabelingLoss
    $N \leftarrow length(\mathcal{D}_{all})$
    $\mathcal{L} = -\frac{1}{N} \sum (w_i \left[y_i \cdot \log \sigma(x_i) + (1 - y_i) \cdot \log(1 - \sigma(x_i))\right]$
    return $\mathcal{L}$

Algorithm 4: Pseudo code for the evaluating the model.

Input

$\mathcal{M}$ Deep Profiler model

$\mathcal{D}_{all}$ pre-processed patient measurements

$\mathcal{D}_{severity}$ severity level

Output

$\mathcal{S}$ Deep Profiler model score

procedure MODELSCORE

  $\mathcal{O}_{labels}, \mathcal{O}_{recon}, \mathcal{O}_{mean}, \mathcal{O}_{var} \leftarrow \text{DEEPPROFILER}(\mathcal{M}, \mathcal{D}_{all})$

  $\mathcal{S} \leftarrow 0$

  for $level \in \{1, 2, 3, 4\}$ **do**

    $\mathcal{S} \leftarrow \mathcal{S} + 0.1 \cdot level \cdot \text{AUROC}(\mathcal{D}_{severity}[level], \mathcal{O}_{labels}[level])$

  return $\mathcal{S}$

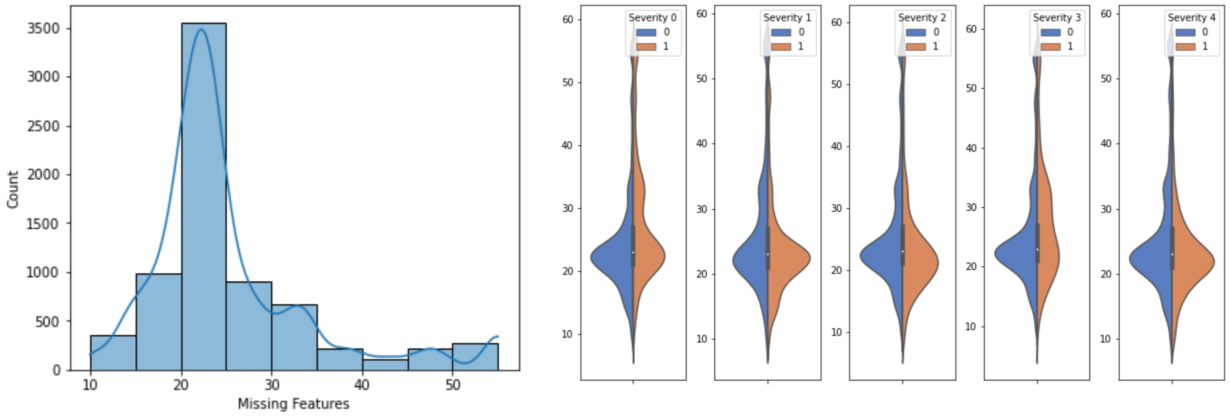

(a) (b)

Figure S1: Studying the distribution of missing data over the (a) shows the histogram of the patient count with number of missing input features (NOTE: the patients with less than 4 features are already removed) (b) shows the distribution of the patients with missing input features by different severity levels.

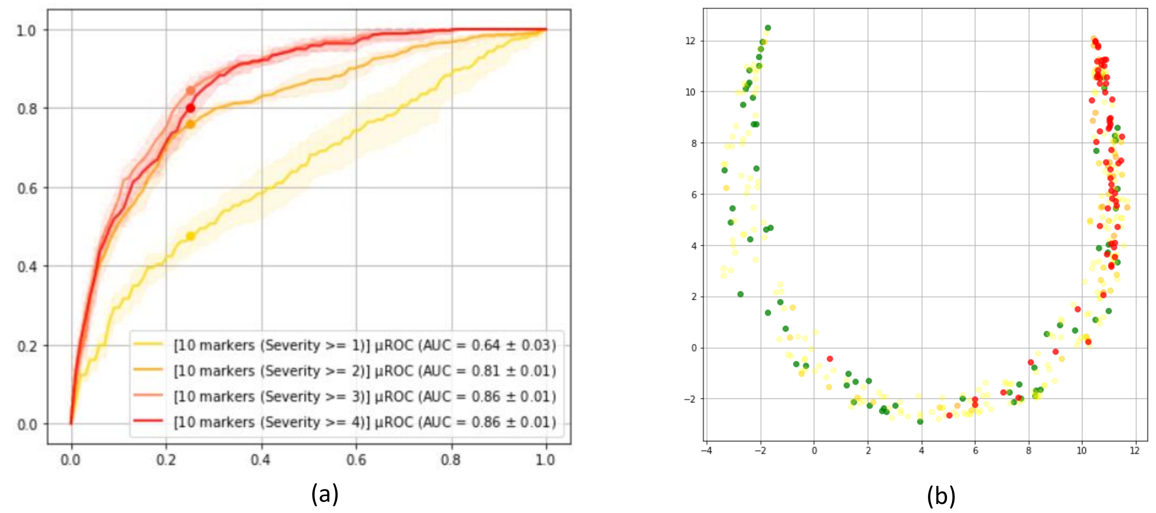

Figure S2: Studying the impact of the missing data on the model (a) shows the performance of the 10-marker deep profiler on a subset of 300 patients in the testing data for which all the 10 measurements were available within first 24 hours of admission. Out of 300 patients, number of patients with severity >=1, >=2, >=3, >=4 is 244, 64, 45 and 36 respectively (b) shows the UMAP visualization of the same 300 patients on the deep profiler latent space for first model in the ensemble.

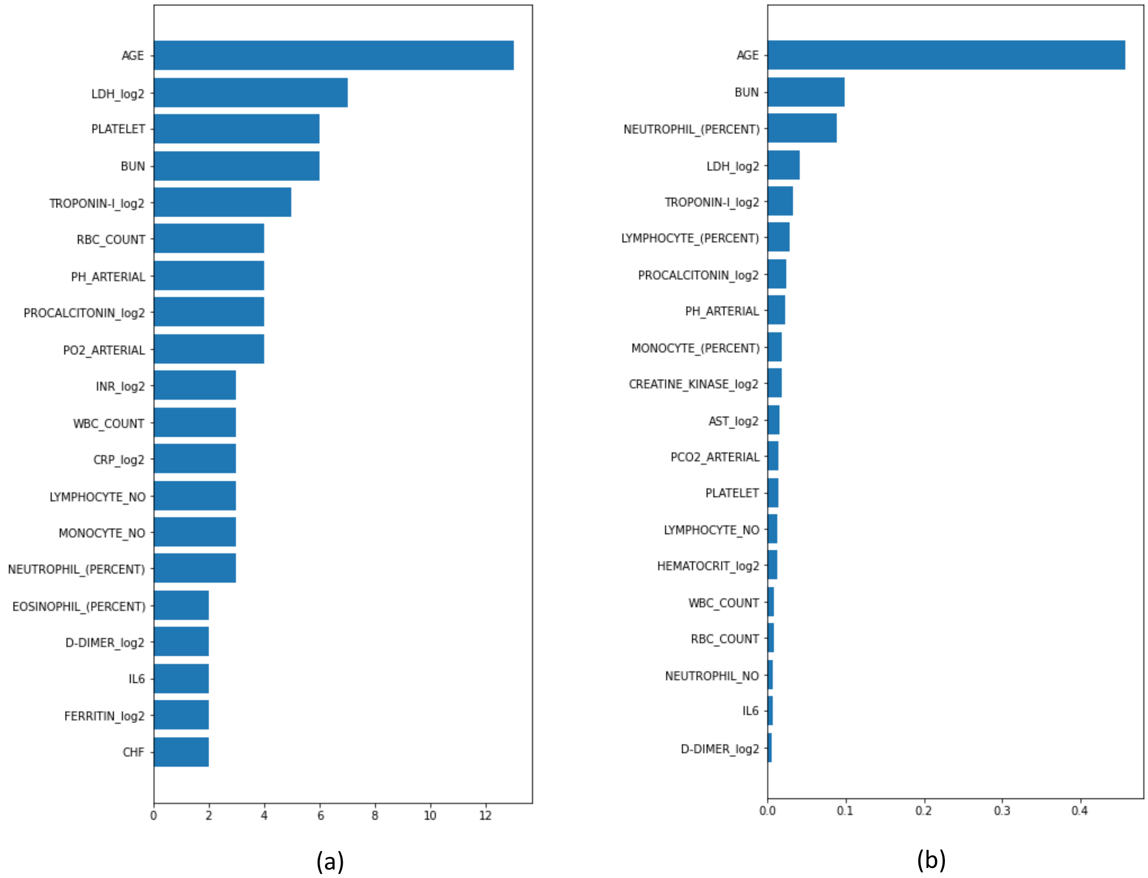

Figure S3: Feature importance of the machine learning models (a) XGBoost (b) Random Forest Regression.
